# Red blood cell extracellular vesicles as robust carriers of RNA-based therapeutics

**DOI:** 10.15698/cst2018.09.155

**Published:** 2018-08-30

**Authors:** Chanh Tin Pham, Xin Zhang, Austin Lam, Minh TN Le

**Affiliations:** 1 Department of Biomedical Sciences, College of Veterinary Medicine and Life Sciences, City University of Hong Kong, 83 Tat Chee Avenue, Kowloon, Hong Kong.

**Keywords:** Cancer, RNA, therapeutics, extracellular vesicles, red blood cells

## Abstract

One of the major challenges of RNA-based therapeutics is the method for delivery of RNA molecules. In a recent article (Nat Commun 9(1):2359), we described a novel delivery platform for RNA-based drugs using red blood cell extracellular vesicles which can efficiently deliver both small and large RNAs to solid and liquid tumours. Our RBCEVs platform features exceptional merits over conventional RNA delivery methods in biosafety, biocompatibility, efficiency, accessibility, and cost effectiveness.

The emerging RNA-based therapeutics are renowned for a wide array of druggable targets, which are difficult to achieve by conventional therapies, with high specificity and great versatility. RNA-based drugs function depends on different mechanisms, such as RNA interference using small-interfering RNAs (siRNAs), inhibition of RNA functions using antisense oligonucleotides (ASOs), and genome editing using the CRISPR-Cas9 system with guide RNAs (gRNAs). The advantages of RNA-based therapeutics boost the translation into various clinical applications, from repairing genetic disorders to fighting against cancers to silencing virus replication. However, there currently exist two major challenges in RNA-based therapeutics: (i) instability of RNA drugs in physiological environment and (ii) difficulty in delivery of RNA drugs. The former can be resolved by chemical modifications to enhance the stability, which has already been tested in clinical trials, whereas the delivery issue remains largely unresolved. Delivery via viruses and/or lipid-based nanoparticles/reagents is suboptimal due to cytotoxicity, immunogenicity, and uncertainty in biosafety.

Extracellular vesicles (EVs), which are secreted by most cell types and represent a natural mode of intercellular communication, are thriving as novel carriers of RNA-based drugs with appreciable biocompatibility. EVs can be isolated from human cells including cancer cells, fibroblasts, epithelial cells, etc., or non-human sources such as plants and bovine milk. RNA-based therapeutic agents such as ASOs, mRNAs, and miRNAs, have been successfully loaded into EVs by passive loading using physical incubation, by active loading using electroporation and sonication, or by transfection of EVs donor cells. The diversity of administration methods of RNAs-loaded EVs ranging from oral administration, intratumoural injection to systematic administration allows the therapeutics to be tailored to maximise the targeting and delivery efficiency to treat various diseases. The beauty of using EVs as RNA-based therapeutics carriers lies in low immunogenicity, high biocompatibility, and natural interaction with target cells in comparison with traditional RNAs delivery strategies. Despite such perks, RNA delivery technology using EVs is hampered by both the difficulty in producing a large amount of homogenous, ultrapure EVs and the risk of horizontal gene transfer due to DNA encapsulated in EVs.

In our recent article, we described a robust RNA drugs delivery platform based on red blood cell extracellular vesicles (RBCEVs), which harbours plenty of advantages over conventional delivery methods. RBCEVs are derived from human red blood cells (RBCs), which lack both mitochondrial and nuclear DNA, thus avoiding the risk of horizontal gene transfer. RBCs have been widely used for blood transfusion over decades, which highlights the extraordinary biosafety and biocompatibility of the filial vesicles, i.e. RBCEVs. Furthermore, RBCEVs can be stored at -80°C for a long period and remain stable and intact, even after multiple freeze-thaw cycles, without affecting the moiety, uptake and RNA loading capacity. Qualities of such can facilitate the storage and transportation of RBCEVs. Last but not least, it is easy to produce ultrapure RBCEVs in a large scale at low cost. RBCs are readily available from human subjects in blood banks and we can treat RBCs with calcium ionophore to induce EV release, then purify intact and ultrapure RBCEVs through series of ultracentrifugation and sucrose cushions. Since no expensive culture medium is needed, the method is rather cost-effective. It is possible to produce about 10^13^ to 10^14^ RBCEVs particles per blood unit with high purity.

In our study, we loaded a variety of RNA-based therapeutics into RBCEVs, used the powerful RBCEVs delivery platform to treat both solid and liquid tumours and observed impressive efficacies. Breast cancer and acute myeloid leukaemia (AML) were used as representatives of solid and liquid tumours in the investigation.

**Figure 1:**
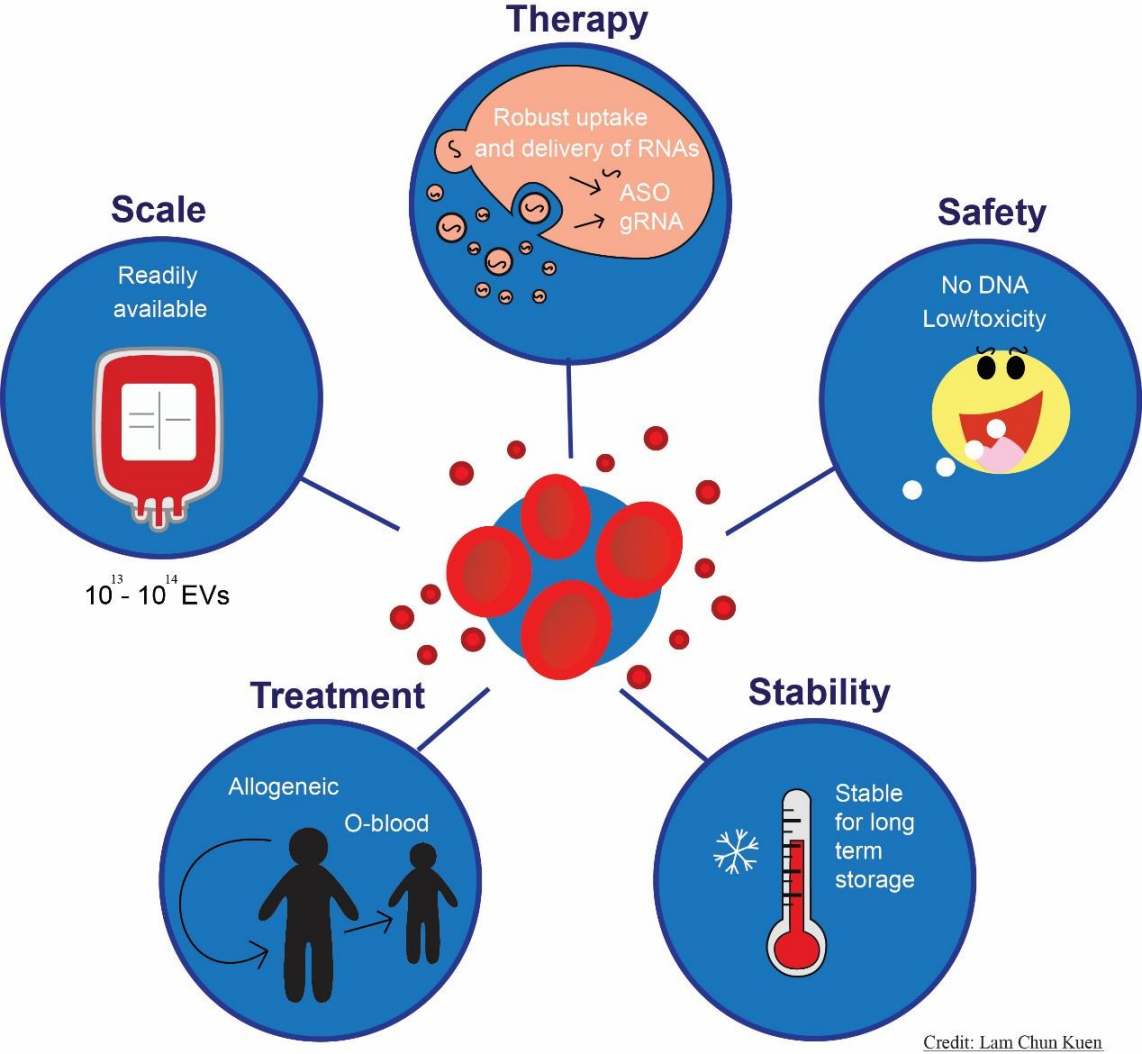
FIGURE 1: Schematic overview of the advantages and potential applications of the red blood cell extracellular vesicles (RBCEVs) delivery system of RNA-based therapeutics.

The human AML cell line MOLM13 is known as a difficult-to-transfect cell line with common RNA delivery vehicles. When using our RBCEVs delivery platform, MOLM13 cells could take up RNA-loaded RBCEVs easily at high efficiency and low toxicity as compared to commercial transfection reagents such as Lipofectamine^TM^ 3000 and INTERFERin. RBCEVs-mediated delivery of miR-125b ASOs resulted in the down-regulation of miR-125b *in vitro*, which led to an increase in the expression level of its target gene *BAK1* and inhibited the proliferation of MOLM13 cells. We observed the same effect in the human metastatic breast cancer MCF10CA1a cells. *In vivo* imaging showed that RBCEVs were taken up by breast cancer cells engrafted in immunodeficient mice with high efficiency. miR-125b ASOs loaded RBCEVs inhibited cancer cell proliferation, suppressed miR-125b expression level and reduced infiltrated cancer cells in AML MOLM13 engrafted mice. In addition, Cas9 mRNA and gRNA were successfully introduced into AML cell line MOLM13 using RBCEVs with agreeable functionality, which demonstrated the capacity of RBCEVs delivery platform in genome editing.

The capacity to load RNA-based drugs including ASOs, Cas9 mRNA and gRNAs or even plasmids has broadened the scope of RBCEVs application to treat diseases with current undruggable targets. Future development of RBCEVs research includes the specific targeting of RBCEVs, which can be achieved by conjugating antibodies or peptides to RBCEVs with the capacity to bind to their target cancer cells specifically. The targeted delivery of RBCEVs will not only enhance the efficacy of RNA drugs, but also significantly reduce side effects on normal tissues, thus weaponizing RBCEVs to treat various diseases.

